# Isolation of *Hermetia illucens* larvae core gut microbiota by two different cultivation strategies

**DOI:** 10.1007/s10482-022-01735-7

**Published:** 2022-04-22

**Authors:** Yina Cifuentes, Andreas Vilcinskas, Peter Kämpfer, Stefanie P. Glaeser

**Affiliations:** 1 Institute of Applied Microbiology, Justus-Liebig University Giessen, IFZ-Heinrich-Buff-Ring 26-32, 35392 Giessen, Germany; 2 Department of Bioresources, Fraunhofer Institute for Molecular Biology and Applied Ecology, Giessen, Germany; 3 Faculty of Agricultural Sciences, Institute for Insect Biotechnology, Nutritional Sciences, and Environmental, Giessen, Germany

**Keywords:** Black soldier fly, Core gut microbiota, Cultivation approach, Dilution-to-extinction, Direct plating, genomic fingerprinting, *Hermetia illucens*

## Abstract

*Hermetia illucens* larvae (black soldier fly larvae, BSFL) convert efficiently organic waste to high quality biomass. To gain knowledge on the specific functions of gut microbes in this process it is a prerequisite to culture members of the core gut microbiota. Two different cultivation strategies were applied here for this purpose, a dilution-to-extinction cultivation and direct plating using six different media to culture aerobic heterotrophic bacteria. A total of 341 isolates were obtained by the dilution-to-extinction cultivation and 138 isolates by direct plating from guts of BSFL reared on chicken feed. Bacterial isolates were phylogenetically identified at the genus level by 16S rRNA gene sequencing (phylotyping) and differentiated at the strain level by genomic fingerprinting (genotyping). The main proportion of isolates was assigned to *Proteobacteria*, *Firmicutes* (*Bacilli*), and *Actinobacteria.* Predominant genera discussed in literature as member of a potential BSFL core gut microbiota, *Providencia, Proteus, Morganella, Enterococcus, Bacillus,* and members of the family *Enterobacteriaceae*, were isolated. A high intra-phylotype diversity was obtained by genomic fingerprinting which was especially enhanced by the dilution-to-extinction cultivation. This study showed that the application of different cultivation strategies including a dilution-to-extinction cultivation helps to culture a higher diversity of the BSFL gut microbiota and that genomic fingerprinting gives a better picture on the genetic diversity of cultured bacteria which cannot be covered by a 16S rRNA gene sequence based identification alone.

**Supplementary Information:**

The online version contains supplementary material available at 10.1007/s10482-022-01735-7.

## Introduction

*Hermetia illucens* larvae, known as black soldier fly larvae (BSFL), can efficiently convert a wide range of organic waste products in high quality biomass with high quality proteins and fats. For that reason larvae are increasingly used as animal feed and human food source (Wang and Shelomi 2017) or for composting (da Silva and Hesselberg 2020). Larval gut microbiota play an important role in the digestion and nutrient provision and for the development, physiology, and the immune system of the larvae (Jeon et al. 2011; Engel and Moran 2013). An important role of the gut microbiota of BLSF during digestion of waste products of animal or human sources is the potential antibacterial activity during digestion which is especially important for the deactivation of pathogenic bacteria present in those substrates (Elhag et al. 2022; Erickson et al. 2004; Liu et al. 2008). The antimicrobial activity of BSFL gut microbes makes their cultivation especially attractive for the research of new antimicrobial compounds (Tegtmeier et al. 2021).

Different studies have already focused on the gut microbiota using mainly molecular cultivation independent methods to explore its community composition (Jeon et al. 2011; Zheng et al. 2013). Changes of the BSFL gut microbiota according to the employed substrate were reported in several publications (Bruno et al. 2019; Gorrens et al. 2021; Jeon et al. 2011; Klammsteiner et al. 2020; Shelomi et al. 2021; Wynants et al. 2019). Some of those studies reported also changes in the bacterial community in the residue of the employed substrates based on the larval digestion process (Bruno et al. 2019; Cifuentes et al. 2020; Wynants et al. 2019; Gold et al. 2020; Shelomi et al. 2021). Shelomi et al. (2021) showed that even through the waste microbiota strongly differed, many taxa were shared in the gut of the differently fed larvae, which indicates the presence of a BSFL specific gut core microbiota. A stable autochthonous bacterial community in the larval gut is one of the general conclusions across all molecular gut microbiota studies. However, there is still no clear consensus among scientists if BSFL have a defined bacterial core gut microbiota and which taxa belong to it (Gorrens et al. 2021). At least 20 genera have been frequently detected in the gut of BSFL reared under different conditions and analysed at different growth stages (Gorrens et al. 2021). As summarized by Gorrens et al. (2021), more than ten studies have detected *Dysgonomonas*, *Enterococcus*, *Morganella,* and *Providencia.* The genera *Klebsiella*, *Clostridium*, *Actinomyces*, *Bacillus*, *Pseudomonas,* and *Ignatzschineria* were all detected in more than three studies in gut samples of BSFL. The high detection frequency of these genera suggested their potential important role in the BSFL gut microbiota. At least some of those taxa could be considered as a core gut microbiota.

High throughput amplicon sequencing of bacterial 16S rRNA genes has been the most employed technique to explore the gut microbiota composition and all possible changes according to the rearing conditions of BSFL. This approach however does not reflect the overall bacterial diversity present below the genus level and cannot cover the genetic diversity present in an environment (Nichols 2007; De Smet et al. 2018). Only few studies are currently available which intended to culture bacteria of the BSFL gut microbiota (Jeon et al. 2011; Kim et al. 2014; Callegari et al. 2020; Shelomi et al. 2021; Gorrens et al. 2021; Tegtmeier et al. 2021). So far, only few different cultivation strategies were applied to culture specific functional members of a potential core gut microbiota. Some studies aimed to culture members of BSFL microbiota that have amylase, cellulase, pectinase, and esterase/lipase activities. For this purpose, Callegari et al. (2020) applied a pre-enrichment step with polymeric substrates as uric acid, filter paper, and carboxymethylcellulose. In contrast, Gorrens et al. (2021) reared BSFL in standard diets supplemented with polymeric compounds as lignin, pectin, keratin, cellulose, and hemicellulose and employed direct plating of serially diluted gut samples on plate count agar. Other studies applied non-selective direct plating approaches using complex media as Luria–Bertani or nutrient agar to isolate abundant heterotrophic gut associated bacteria from BSFL reared in different diets (Jeon et al. 2011; Shelomi et al. 2021). Tegtmeier et al. (2021) used an extended spectrum of cultivation conditions to study the diversity of physiological properties of endogenous BSFL bacteria. They used different buffers or broth media to prepare dilution series of BSFL homogenates which were plated on different selective and non-selective agar media. In contrast to other studies both, oxic and anoxic incubation conditions were applied. They isolated similar bacterial taxa as in the other studies but recovered an expanded range of bacterial diversity including some representatives of the less-abundant phylum *Actinobacteria* and potential new genera of anaerobic *Clostridiales*.

The aim of this study was to generate a culture collection of abundant aerobic heterotrophic bacteria representing members of the core gut microbiota from BSFL larvae as a starting point to investigate the genetic potential of the BSFL core gut microbiota. To extend the diversity of cultured gut bacteria a dilution-to-extinction enrichment cultivation approach was applied beside a standard direct plating approach. The dilution-to-extinction enrichment cultivation was not yet applied to the cultivation of bacteria from the gut of BSFL larvae. This approach was originally introduced by Button et al. (1993) for the cultivation of typical small abundant but slow growing oligotrophic marine bacteria which are normally outcompeted by fast growing bacteria by the direct plating cultivation or which could not directly grow on an agar plate surface due to the higher oxygen tension there. For this approach, suspensions of environmental bacteria were directly serially diluted in an enrichment medium which based on filtered marine water which was supplemented with low amounts of complex substrates as peptone (Button et al. 1993). After several weeks of incubation, the enrichment cultures of the highest positive dilutions were streaked on agar plates containing the same media used for the enrichment. This approach was later described as a miniaturized dilution-to-extinction enrichment (Hoefman et al. 2012). We applied this approach to culture especially abundant, but slow growing bacteria from the gut microbiota or to culture gut symbionts that may have a problem to grow directly on an agar surface due to the high oxygen tension at the air agar surface interface. In the second approach, a standard direct plating approach, suspensions of the gut microbiota were serially diluted in sterile 0.9% (w/v) sodium chloride and plated directly on different agar media. A broad range of morphological different colonies was selected for the subsequent analyses. All obtained isolates (pure cultures) were identified by 16S rRNA gene sequencing (phylotyping) and differentiated at the strain level (intra-phylotype diversity) by genomic fingerprinting through BOX- and (GTG)_5_-PCRs (genotyping).

## Materials and methods

### Sampling and gut dissection

Black solder fly larvae (BSFL) were supplied by the Bio.S Biogas company (Grimma, Germany). The larvae were reared for four weeks at 25 °C on commercial chicken feed.

Larvae were incubated at − 20 °C for 10 to 15 min to inactivate the larvae. Larvae were then surface-sterilized by incubation in 5% (v/v) sodium hypochlorite for 1 min and washed shortly in phosphate-buffered saline (PBS pH 7.0) before dissection of the guts. The dissection was carried out under the stereomicroscope on a sterile glass slide under oxic conditions. Each extracted gut was cleaned by washing in autoclaved pure water. The guts were collected in sterile 50 ml polypropylene tubes. Each of three sample replicates contained a pool of ten guts. Gut samples are shown in Supplementary Fig. S1. The guts were resuspended in 10 ml of 0.22 µm-filter-sterilized 0.2% (w/v) tetra-sodium-pyrophosphate (TSPP) and a mechanical treatment in a Stomacher ® 80 Biomaster (Seward Limited) was used two times for 1 min and 30 beats/s to detach bacterial cells from the gut material. The bacterial cell suspensions were used to culture core gut bacteria.

### Cultivation

Each of the resuspended gut microbiota samples (three replicates, R1–R3) were used as inoculum for the dilution-to-extinction cultivation and for the direct plating approach. The cultivation approaches were started immediately after the gut microbiota was harvested. The procedure used for the direct plating approach started with a tenfold serial dilution of 1 ml of the cell suspensions in 0.9% (w/v) sodium chloride (NaCl). The dilution series was set up in glass tubes until 10^–4^. Cell suspensions were always mixed by vortexing. An aliquot of each dilution (100 µl) was plated (from 10^0^ to 10^−4^) on brain heart infusion (BHI) agar (Difco), lysogeny broth (LB Lennox, ROTH) agar, trypticase soy (TS) (Difco) agar (Roth), Reasoner’s 2A (R2A) agar (Oxoid), and Mueller–Hinton (MH) agar (Roth), respectively. All plates were incubated for 2 days under oxic conditions in the dark at 25 °C.

The dilution-to-extinction enrichment cultivation was performed in 96-well plates (Greiner BIO-ONE) using different media, half-concentrated R2A (½ R2A), R2A, LB, and TS broth.

The 96-well plate was pre-loaded with 180 µl sterile broth per well. In the first row the broth media were inoculated with 20 µl of the gut cell suspensions. Each of the three gut suspensions were used as a biological replicate once for each applied medium as inoculum. The inoculated media in the first wells were serially diluted from A to H using a multichannel pipette. Cell-medium suspensions were mixed by pipetting up/down for 10 times and 20 µl of the suspensions were transferred to the next well, where mixing was repeated. The serial dilutions per sample contained eight dilution steps. Plates were closed with sterile plastic lids and incubated for 4 weeks at 25 °C in a closed container with a wet tissue to avoid the evaporation of the media. After this period of incubation, 10 µl were removed from the individual wells and were streaked on agar plates with the same media as used for the enrichment cultivation. The remaining samples in the 96-well plate were conserved at − 20 °C after the addition of glycerol in a final concertation of 20% (v/v). Agar plates were incubated for 2–3 days at 25 °C. Colonies with different morphologies were selected from the different agar plates. One to two inoculation loops full of fresh biomass of purified strains were suspended in 1.4 ml u-bottom push cap tubes (Micronic, Netherlands) with 250 or 500 μl Gibco newborn calf serum (NBCS, ThermoFisher Scientific) and stored at − 20 and − 80 °C for long-term preservation. In parallel one inoculation loop full of fresh biomass was resuspended in 100 or 500 μl molecular grade water (Roth) to obtain cell lysates using the freeze–thaw method (Schauss et al., 2015).

### Genotyping

Genotypic differentiation (genotyping) of isolated bacteria was performed by genomic fingerprinting using BOX-PCR with primer BOX1AR (5′-CTA CGG CAA GGC GAC GCT GAC G-3′) (Versalovic et al. 1994). Analyses were performed according to Glaeser et al. (2013). For isolates which were identified as members of the genera *Enterococcus* and *Mammaliicoccus* (see below) BOX-PCR gave insufficient results, hence (GTG)_5_-PCR was used according to Glaeser et al. (2016) with primer (GTG)_5_ (5’-GTG GTG GTG GTG GTG-3’) (Versalovic et al. 1994). Genomic fingerprint patterns generated by agarose gel electrophoresis (Glaeser et al. 2013) were compared in BioNumerics version 8.0 (Applied Maths N.V.). A similarity matrix comparing the individual fingerprint patterns was calculated with the Pearson product–moment correlation (Pearson correlation). Cluster analysis was performed with the unweighted pair group method with arithmetic mean (UPGMA) with 1% position tolerance and 0.5% optimization. Isolates were assigned to one genotype if they shared identical genomic DNA fingerprint patterns.

### Phylogenetic identification by partial 16S rRNA gene sequencing (phylotyping)

Representative isolates of each genotype were identified by partial 16S rRNA gene sequencing. The primer system 8F (5′-AGA GTT TGA TCC TGG CTC AG-3′) and 1492R (5′-ACG GCT ACC TTG TTA CGA CTT-3′) (Lane 1991) was used to amplify the 16S rRNA gene as described previously (Aydogan et al. 2020). PCR products were sequenced with the Sanger method using primers 27F (5′-GAG TTT GAT CMT GGC TCA G-3′) and E786F (5′-GAT TAG ATA CCC TGG TAG-3′). PCR product purification and sequencing reactions were performed by LGC Genomics (Berlin, Germany).

DNA sequences were corrected manually using MEGA 7.0 (Kumar et al. 2016) based on the electropherograms. Ambiguous positions at the 5′ and 3′ ends of the sequences were removed. A first identification of the phylogenetic affiliation of the strains was done by BLAST analysis against the EzBioCloud database (Yoon et al. 2017) resulting in 16S rRNA gene sequence similarities of closely related type strains included in the database. Subsequently the sequences were added to the phylogenetic tree containing type strain sequences using the All-Species Living Tree Project (LTP) (Yarza et al. 2008), database releases 132 (June, 2018). Analysis was performed in ARB release 5.2 (Ludwig et al. 2004). The 16S rRNA gene sequences were first aligned with SINA v1.2.9 according to the SILVA seed alignment (http://www.arb-silva.de) (Pruesse et al. 2012) and imported into the LTP database tree using the parsimony quick add marked sequences tool implemented in ARB. The partial sequences were thereby placed into the database tree without changing the overall tree topology. The alignment of all sequences considered for phylogenetic analysis was checked manually considering the secondary structure of the 16S rRNA. A subtree containing the added partial 16S rRNA gene sequences and a selection of reference strains from the database tree was exported. For the definition of phylotypes additional phylogenetic analyses were performed in MEGA 7.0 using partial sequences of the isolates and type strain reference sequences. Sequences were aligned with ClustalW implemented in MEGA 7.0. Analyses were performed separately for different bacterial phyla. Alignments were corrected manually. Pairwise sequence similarities were determined in MEGA 7.0 with the p-distance method without using evolutionary models. Phylogenetic trees for phylotype assignment were constructed with the Neighbour joining method using the Jukes Cantor Correction model. Trees were calculated considering 100 repetitions (bootstrap analysis). Isolates were assigned to phylotypes which were defined by the formation of monophyletic clusters within the phylogenetic tree which was supported by high bootstrap values. Sequences present within one cluster shared at least 99.0% partial 16S rRNA gene sequence similarities among each other.

### Seriation analysis

Seriation analysis was performed in PAST4.04 (Hammer et al. 2001) to compare the occurrence patterns of phylotypes within the different replicates cultured by the two different cultivation strategies and in/on the different media (Franco et al. 2020). Analyses were based on an absence-presence (0/1) matrix using the algorithm described by Brower and Kile (1988).

## Results

### Overview of cultured bacteria (phylotyping)

Bacterial growth was obtained by both cultivation strategies in, or on all applied media for the investigated BSFL gut samples (three independent pools of guts of ten larvae). All wells of the serially diluted samples (up to 10^–8^) in the dilution-to-extinction approach and all plated dilutions (up to 10^–5^) showed bacterial growth. In total a collection of 479 isolates (341 from the dilution-to-extinction approach and 138 from direct plating) were cultured from the different agar media. The isolates represented the morphological diversity of abundant colony morphologies grown on the agar plates. Based on the 16S rRNA gene sequence analyses the isolates were assigned to ten different genera of *Proteobacteria* (*Stenotrophomonas, Pseudomonas, Alcaligenes, Proteus, Providencia, Morganella, Serratia, Klebsiella/Enterobacter,* and *Brucella*) and four different genera of *Firmicutes,* all of the order *Bacilli (Bacillus, Enterococcus, Mammaliicoccus,* and *Lysinibacillus*).

**Fig. 1:**
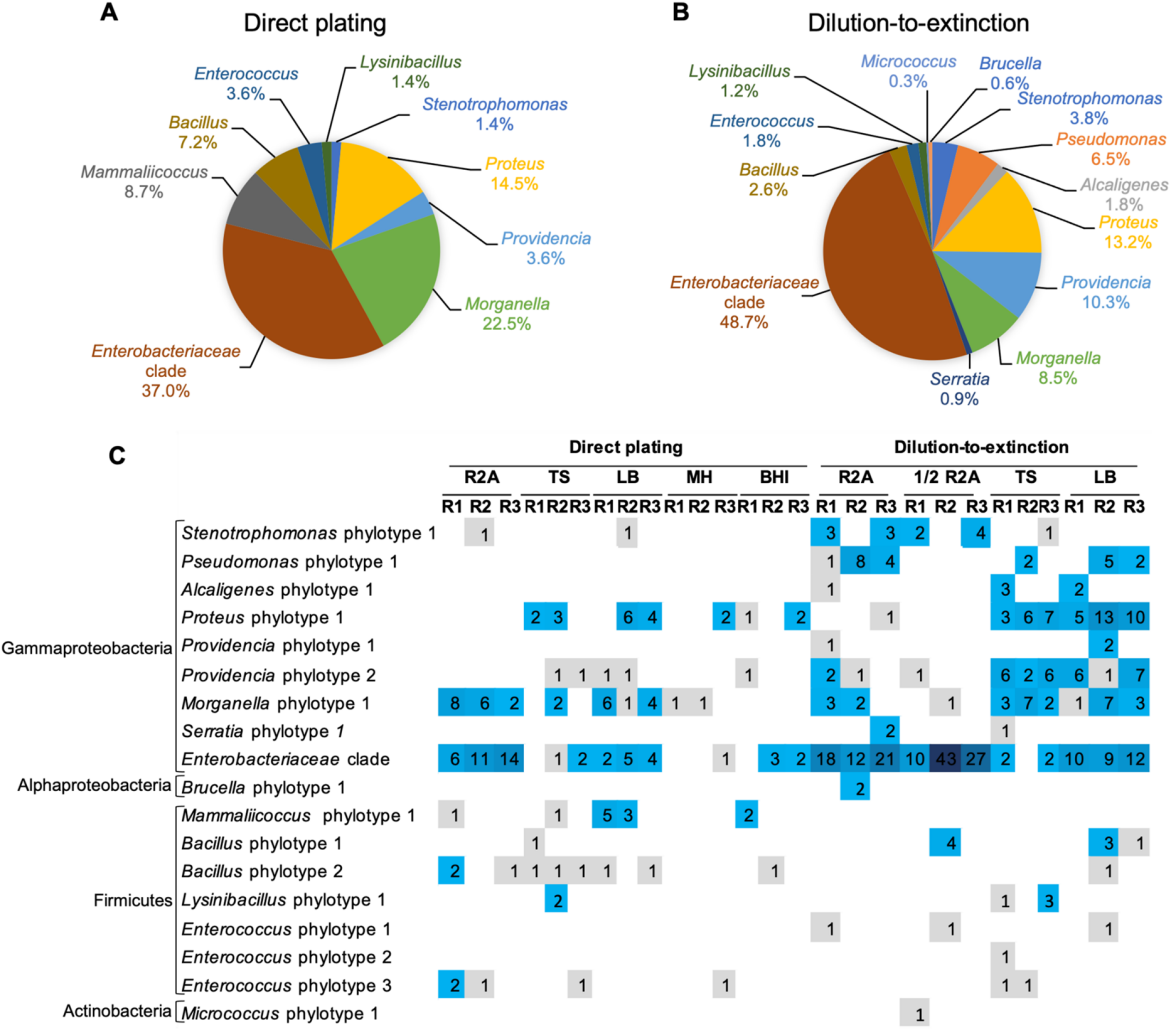
Overview of the bacterial genera cultured from gut samples of black solder fly larvae (BSFL) by the use of two different cultivation methods, dilution-to-extinction enrichment cultivation and direct plating cultivation. **A**, **B** Genera distribution according to the detected phylotypes from all isolates cultured by direct plating (**A**) and dilution-to-extinction cultivation (**B**). Values were calculated based on a total of 138 isolates for the direct plating (**A**) and 341 isolates for the dilution-to-extinction cultivation (**B**). **C** Phylotype (defined based on 16S rRNA gene sequence data, Fig. 2) distribution according to the employed media (R2A, ½ R2A, TS, LB) and cultivation approaches. The numbers in **C** represent the numbers of studied isolates derived from individual colonies. R1-R3 represent studied replicates. Each replicate represented a pool of ten BSFL guts

Isolates identified as *Enterobacteriaceae* (closely related to *Enterobacter/Klebsiella*) represented the largest proportion of isolates by both cultivation strategies (49% and 37% by the dilution-to-extinction and direct plating cultivation, respectively) (Fig. 1). The most abundant taxa cultured by the dilution-to-extinction approach were *Proteus* (13%), *Pseudomonas* (6%), *Providencia* (10%), and *Morganella* (8%) and by the direct plating approach *Morganella* (22%), *Proteus* (14%), *Mammaliicoccus* (9%), and *Bacillus* (7%). Other detected taxa occurred with a low relative abundance (0.3–4%).

**Fig. 2:**
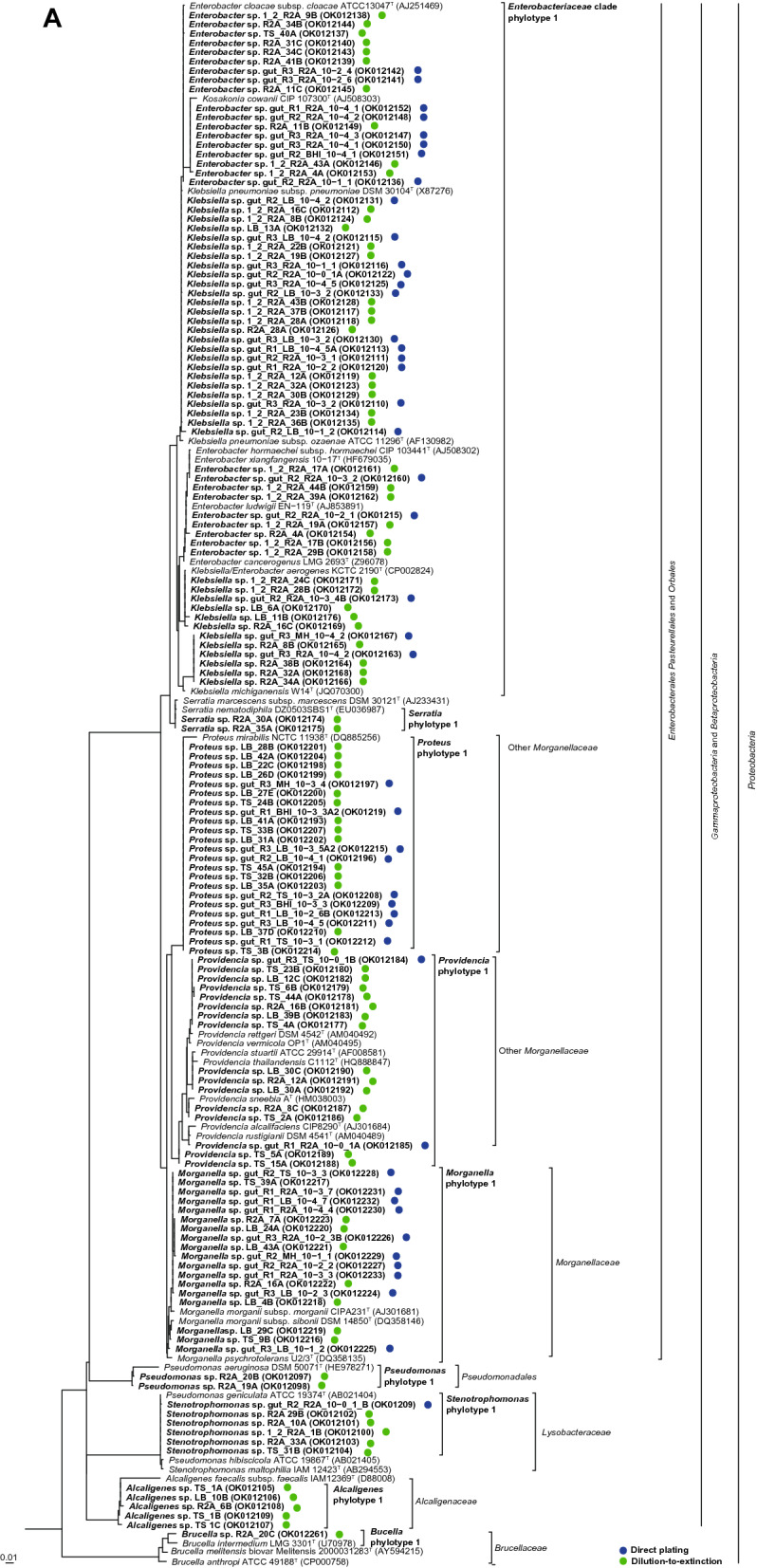

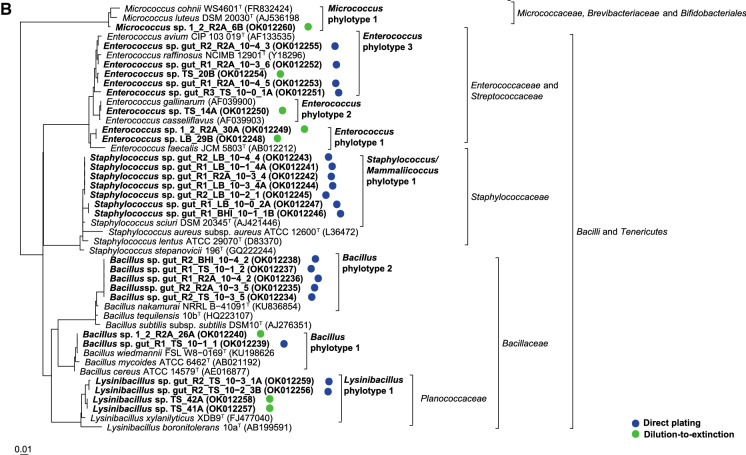
Placement of the 16S rRNA gene sequenced isolates into the type strain tree provided in the LTP database releases 132 (June, 2018) without changing the tree topology. **A** Proteobacteria. **B** Other phyla. Analysis was performed in ARB. Circles behind the isolate numbers indicate the applied cultivation method. Acc. numbers of the 16S rRNA gene sequences are given in parenthesis. Blue and green dots represent the isolates obtained from direct plating and dilution-to-extinction approaches, respectively. (Color figure online)

Within the different genera, isolates were differentiated into up to six different phylotypes per genus which showed distinct clusters in the phylogenetic trees (Fig. 2, Supplementary Fig. S2a–c). A total of 18 different phylotypes were detected throughout this study. They all shared very high 16S rRNA gene sequence similarities (> 99%) to type strains of single or partially several related species. Due to the high 16S rRNA gene sequence similarity among members of the *Enterobacteriaceae* a phylotype differentiation was not possible for the isolates closely related to *Enterobacter/Klebsiella*. The strains were summarized as an *Enterobacteriaceae* clade. *Providencia* isolates represented two distinct phylotypes. Isolates of the first phylotype clustered with the type strain of *Providencia stuartii* and isolates of the second phylotype with several other *Providencia* species type strains with *Providencia rettgeri* as closest related one. All isolates identified as members of the genus *Proteus* belonged to one phylotype and clustered with the type strain of *Proteus mirabilis*. *Morganella* isolates formed one large cluster and were assigned to one phylotype. The isolates showed highest 16S rRNA gene sequence similarities to the type strains of *Morganella morganii* subsp. *morganii* and *Morganella morganii* subsp. *sorbia*. Isolates assigned to the genera *Serratia*, *Alcaligenes*, *Stenotrophomonas*, and *Mammaliicoccus* were always assigned to one phylotype and closest related to type strains of the species *Serratia marcescens, Stenotrophomonas maltophilia*, *Pseudomonas aeruginosa*, *Alcaligenes faecalis,* and *Mammaliicoccus sciuri* (formerly *Staphylococcus sciuri).*

*Bacillus* and *Enterococcus* isolates were assigned to two and three different phylotypes. These isolates were closely related to the type strains of *Bacillus cereus, Bacillus siamensis, Enterococcus faecalis, Enterococcus mediterraneensis,* and *Enterococcus durans/ Enterococcus avium,* respectively.

### Phylotype distribution

Seriation analysis showed that six different phylotypes including phylotypes of the genera *Pseudomonas, Alcaligenes, Providencia, Serratia, Brucella, Micrococcus,* and *Enterococcus* occurred exclusively among the isolates cultured by the dilution-to-extinction cultivation while only the *Mammaliicoccus* phylotype was only cultured by the direct plating approach (Fig. 2).

### Genotyping

**Fig. 3:**
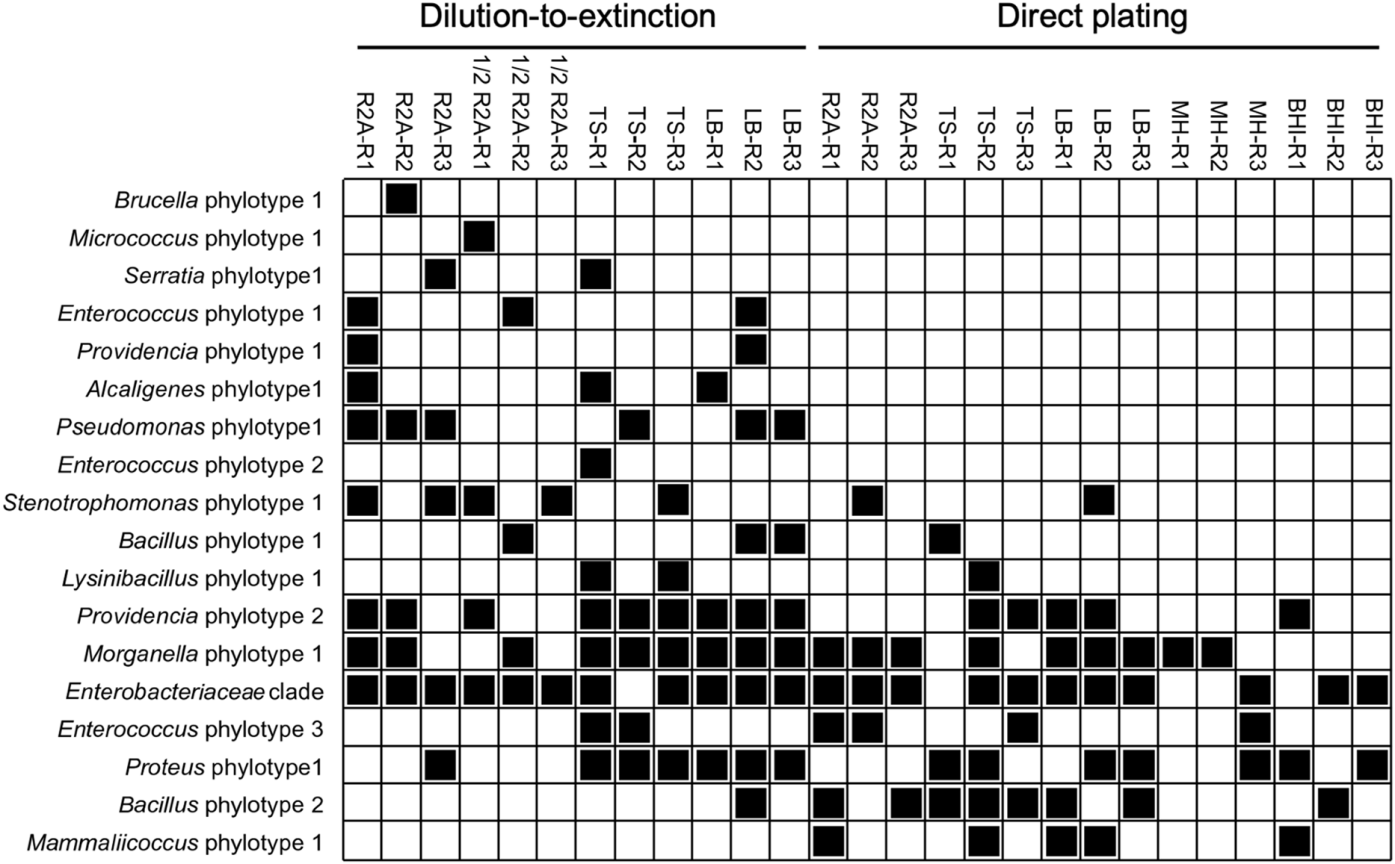
Seriation analysis based on an absence-presence (0/1) matrix illustrating the presence of each phylotype/clade in all different media through miniaturized dilution-to-extinction and direct plating approach

Genomic fingerprinting, BOX and (GTG)_5_-PCR based patterns, enabled the comparison of the isolated bacteria at a higher genetic resolution (strain level). The analysis showed that the genetic diversity of the cultured bacteria was higher than obtained by the phylotype based differentiation. Genomic fingerprinting indicated a high intra-phylotype based genetic diversity (Fig. 3).

**Fig. 4:**
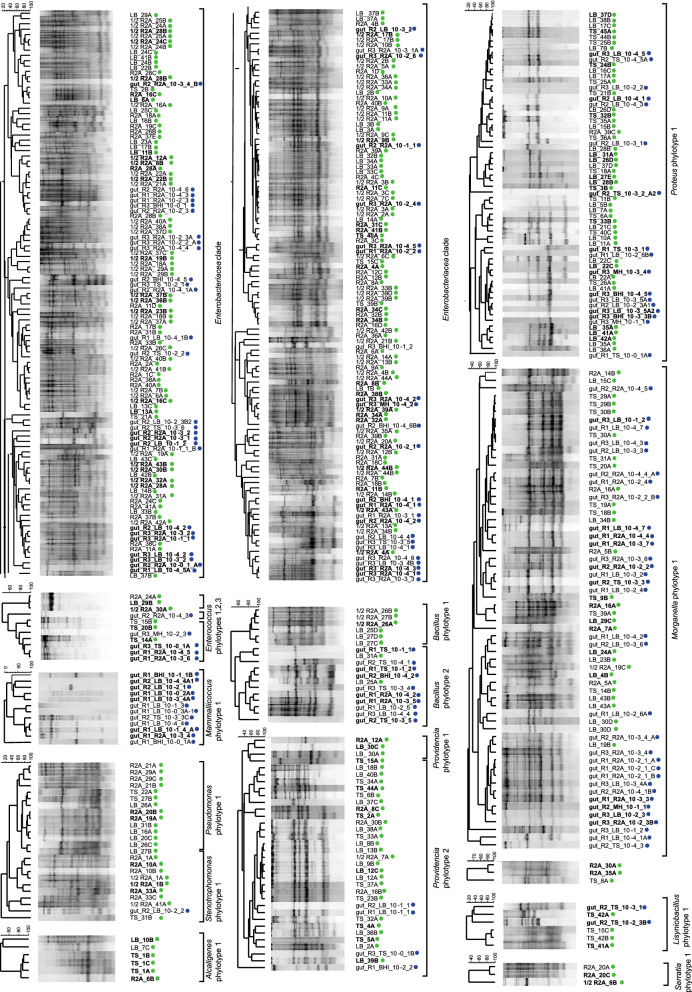
Genotyping of all identified phylotypes isolated from BSFL gut microbiota, based on fingerprint pattern obtained by genomic fingerprinting using primers targeting BOX and (GTG)_5_ repetitive elements. (GTG)_5_-PCR was employed exclusively with *Mammaliicoccus* and *Enterococcus* phylotypes. Cluster analysis was performed in BioNumerics (Applied Maths) using UPGMA clustering, based on a dissimilarity matrices generated by the Pearson correlation. Bold isolates were identified through 16S rRNA gene sequencing. Blue and green dots represent the isolates cultured  by direct plating and dilution-to-extinction cultivation. (Color figure online)

The highest genotype diversity was determined for isolates assigned to the *Enterobacteriaceae* clade and the genera *Providencia, Enterococcus,* and *Morganella* (Fig. 4). Only isolates assigned to the genera *Mammaliicoccus*, *Alcaligenes,* and *Serratia* showed for all isolates identical genomic fingerprint patterns within a phylotype, indicating genetically identity or clonality. Some genotypes were isolated by both cultivation strategies and from different media, while others were only cultured from individual cultivation strategies and individual media.

### Specific observations

A high abundance of swarming bacteria, overgrowing the colonies of other bacteria, were obtained by the direct plating approach on TS, MH, and LB agar, but not on R2A and half concentrated R2A agar. On TS agar some bacterial colonies inhibited the growth of the swarming bacterium in the area around the colonies (Supplementary Fig. S3). The swarming bacteria were identified as *Proteus* spp. Genomic fingerprinting indicated that different *Proteus* spp. seemed to be present in the BSF gut (Fig. 4). The *Proteus*-inhibiting bacteria were identified as *Bacillus* spp. closely related to the type strains of different *Bacillus* species including *Bacillus siamensis, Bacillus amyloliquefaciens,* and *Bacillus velezensis*. The 16S rRNA gene sequence similarities to those type strains were 99.89–100.0%.

## Discussion

The parallel application of two different strategies for the cultivation of aerobic heterotrophic bacteria from BSFL guts enabled the identification of a high diversity of bacteria assigned to genera discussed as members of a potential BSFL core gut microbiota (Gorrens et al. 2021). The application of the two cultivation strategies enhanced thereby the genetic diversity of the cultured bacteria with more different phylotypes and genotypes.

In our previous study we investigated the gut microbiota of BSFL fed with the same substrate by a culture-independent 16S rRNA gene amplicon sequencing based approach (Cifuentes et al. 2020). In that study nine genera were proposed as members of a potential gut core microbiota, *Providencia*, *Enterobacter*, *Bacillus*, *Enterococcus*, *Klebsiella, Morganella*, *Dysgonomonas*, *Lachnospiraceae,* and *Actinomyces.* This was in accordance with other studies that investigated the BSFL gut microbiota (Gorrens et al. 2021).

Here we were able to culture representatives of six of those genera, *Providencia*, *Enterobacter*, *Bacillus*, *Enterococcus*, *Klebsiella*, and *Morganella*. Representatives of the other three genera, *Dysgonomonas*, *Lachnospiraceae,* and *Actinomyces*, could not be isolated. One reason may be that only aerobic cultivation conditions were applied. Members of respective genera may require anaerobic cultivation conditions (Pramono et al. 2015; Feingold and Meislich 2018). Similar observations were made in the study of Tegtmeier et al. (2021). *Dysgonomonas* were not mentioned at all in their study. However, a successful isolation of *Dysgonomonas* under anaerobic cultivation conditions was for example reported for cockroaches (Vera-Ponce de León et al. 2020). Tegtmeier et al. (2021) pointed out that they were also not able to culture *Lachnospiraceae* and *Actinomyces* however respective taxa were detected in the culture independent community analysis of the gut microbiota. None of the other studies that cultures BSFL gut bacteria cultured those genera.

Bacteria assigned to six more genera that were not defined as potential core gut microbiota but detected through 16S rRNA gene amplicon sequencing of the BSFL gut microbiota of larvae from the same provider and fed with the same diet (Cifuentes et al. 2020) were also isolated here. The isolates were identified as members of the genera *Proteus*, *Alcaligenes*, *Mammaliicoccus*, *Micrococcus*, *Stenotrophomonas,* and *Pseudomonas*.

We performed a comparison to other studies that isolated BSFL gut microbes (Table 1). Based on the information given in the different publications bacteria assigned to 37 genera were cultured from BSFL guts. Members of two genera (*Proteus* and *Enterococcus*) were isolated in five studies including ours. Four genera (*Providencia, Morganella, Klebsiella*, and *Bacillus*), were cultured in four different studies including ours. Seven genera were isolated in three different studies among those five which were detected also in our study (*Enterobacter, Alcaligenes, Stenotrophomonas, Mammaliicoccus, Brucella*). The number of different detected genera ranged from just three by Jeon et al. (2011) and Shelomi et al. (2021) to up to 27 genera in the study of Tegtmeier et al. (2021). The higher number of taxa cultured by Tegtmeier et al. (2021) compared to our study (15 different detected genera) may be linked to the inclusion of anaerobic cultivation conditions by Tegtmeier et al. (2021).

**Table 1:** Comparison of detected genera mentioned in studies which cultured gut bacteria from BSFL

Genera	This study	Jeon et al. (2011)	Callegari et al. (2020)	Shelomi et al. (2021)	Gorrens et al. (2021)	Tegtmeier et al. (2021)	No. of studies
*Proteus*	x	x	x		x	x	5
*Enterococcus*	x		x	x	x	x	5
*Providencia*	x		x		x	x	4
*Morganella*	x		x		x	x	4
*Klebsiella*	x		x		x	x	4
*Bacillus*	x	x	x			x	4
*Enterobacter*	x		x		x		3
*Alcaligenes*	x		x			x	3
*Stenotrophomonas*	x		x		x		3
*Mammaliicoccus*	x		x			x	3
*Brucella*	x		x			x	3
*Escherichia*			x		x	x	3
*Citrobacter*			x	x		x	3
*Pseudomonas*	x		x				2
*Micrococcus*	x		x				2
*Lysinibacillus*	x					x	2
*Acinetobacter*			x	x			2
*Vagococcus*			x			x	2
*Bordetella*			x			x	2
*Leucobacter*			x			x	2
*Myroides*			x			x	2
*Paenicaligenes*		x				x	2
*Sphingobacterium*					x	x	2
*Serratia*	x						1
*Pantoea*			x				1
*Koukoulia*						x	1
*Corynebacterium*						x	1
*Rhodococcus*						x	1
*Dietzia*						x	1
*Kocuria*						x	1
*Brevibacterium*						x	1
*Microbacterium*						x	1
*Clostridium*						x	1
*Neglecta*						x	1
*Chryseobacterium*					x		1
*Rummeliibacillus*					x		1
*Glutamicibacter*					x		1
Genera/study	15	3	21	3	12	27	

Members of *Enterobacteriaceae (Klebsiella*/*Enterobacter)*, *Proteus*, *Providencia*, *Morganella,* and *Enterococcus* were represented by several isolates in our study. Representatives of those genera were isolated in different studies from BSFL larvae reared under different rearing conditions and using different cultivation strategies (Callegari et al. 2020; Gorrens et al. 2021; Tegtmeier et al. 2021). Only some hints are available for the function of those bacteria in the BSFL gut. Feeding experiments with different substrates and in parallel performed gut microbiota studies indicated their role in the degradation of polymeric substances (Jiang et al. 2019; Gold et al. 2020; Mazza et al. 2020; Gorrens et al. 2021; Schreven et al. 2021). The use of different cultivation or enrichment media indicated the presence of specific enzymatic activities in respective taxa (Callegari et al. 2020; Gorrens et al. 2021; Tegtmeier et al. 2021).

The addition of BSFL isolates to the feed of BSFL demonstrated that members of the BSFL gut microbiota, as bacteria of the genus *Proteus* enhance the BSFL biomass production (Mazza et al. 2020; Sontowski and van Dam 2020). BFSL based vermicomposting studies indicated the important role of *Enterococcus* and *Providencia* strains in the BSFL gut due to their complex carbohydrate-degrading enzymes and nitrogen, hydrogen, and sulphur metabolism (Jiang et al. 2019; Gold et al. 2020; Gorrens et al. 2021). Further studies indicated the potential capability of *Providencia* to digest xylan (Sontowski and van Dam 2020) and an important role for enterococci during the starvation process in BSFL (Yang et al. 2021). Despite the fact that *Morganella* was reported as pathogen by some *Diptera* spp. (Sontowski and van Dam 2020), members of the genus are frequently found in high abundance in the gut microbiota of healthy appearing BSFL (Table 1).

Different studies indicated that *Providencia* and *Proteus* are involved in the hydrolysis of urea (Gold et al. 2020; Klammsteiner et al. 2020). *Klebsiella* and *Enterobacter* showed a strong correlation to nitrogen fixation in *Diptera* species which significantly contributed to the nitrogen uptake of the larvae (Behar et al. 2005). Specific pectinolytic activities have been recently attributed to *Klebsiella* spp. isolated from the BSFL gut (Callegari et al. 2020; Gorrens et al. 2021). Knowledge on the function of *Dysgonomonas* spp. is only available from isolates cultured from other insects. Those studies indicated an important role of *Dysgonomona*s in the degradation of starch, pectin and cellulose due to the abundance of carbohydrate-active enzyme (CAZyme)-coding genes (Vera-Ponce de León et al. 2020).

Compared with the other three large collections of the BSFL gut isolates (Callegari et al. 2020; Gorrens et al. 2021; Tegtmeier et al. 2021), Callegari et al. (2020) and Tegtmeier et al. (2021), have the most shared phylotypes with our study. The shared phylotypes correspond to genera with low abundance in our study as *Mammaliicoccus*, *Alcaligenes*, *Stenotrophomonas, Brucella,* and *Bacillus.* Members of the genus *Bacillus* and *Stenotrophomonas* were present in high abundance in the study of Callegari et al. (2020). Their study indicated that *Bacillus* spp. are involved in the breakdown of cellulose and starch while *Stenotrophomonas* spp. are involved in the digestion of casein due to pectinase and lipase activities.

Some of the genera that were cultured exclusively by the dilution-to-extinction approach in our study, like *Micrococcus* and *Pseudomonas,* were also cultured by Callegari et al. (2020) and detected previously by our culture-independent 16S rRNA gene amplicon sequencing approach (Cifuentes et al. 2020). The fact that in three studies that cultured bacteria from the gut of BSFL, only Callegari et al. (2020) and the present study have successfully isolated *Pseudomonas* indicates that enrichment methods may be required for a successful cultivation of gut associated *Pseudomonas.* In general, *Pseudomonas* strains can be easily cultured on different complex media (Palleroni 2015). However, here and in another study of other insect guts (unpublished data) we obtained specific subgroups of *Pseudomonas* which were only culturable by the application of the dilution-to-extinction enrichment cultivation in liquid medium and extended time of incubation. The reason for this selective cultivability of some *Pseudomonas* was not further studied yet.

The bacterial collection from Tegtmeier et al. (2021) was obtained from BSFL of the same provider as the BSFLs studied here. This may be one reason why a high proportion of identical taxa were isolated in both studies. Among those members of the genus *Lysinibacillus.* Members of this genus were recently correlated to an increase of the BSFL weight if they were added as food supplement (Mazza et al. 2020; Schreven et al. 2021).

*Serratia* spp. were cultured exclusively in our study from BSFL guts (Table 1). Strains of this genus were in our study exclusively cultured by the dilution-to-extinction cultivation. *Serratia* spp. were also cultured from gut samples of *Diptera* spp. (Jang and Nishijima 1990; Zurek et al. 2000). They are discussed to be on the one hand insect pathogens (Grimont and Grimont 1978), but also present in healthy insects indicating a non-pathogenic interaction with those (Jang and Nishijima 1990; Zurek et al. 2000). *Serratia* spp. in contrast are one of the most frequently isolated genera from cockroaches (Guzman and Vilcinskas 2020). Studies summarized by Guzman and Vilcinskas (2020) also indicated that they are normal members of the gut microbiota of cockroaches because *Serratia* were isolated from healthy, sick, and dead cockroaches, respectively.

Only a part of the studies that cultured BSFL gut microbes provided 16S rRNA gene sequence data of the identified bacteria which made a phylogenetic diversity based study problematic.

Beside our study only Tegtmeier et al. (2021) looked in some extend also to a higher genetic resolution including genomic fingerprinting. Phylotyping (16S rRNA gene sequence based analysis) and genotyping (genomic fingerprinting using e.g. BOX-PCR) as performed here showed a higher genetic diversity of BSFL gut bacteria than obtained by 16S rRNA gene amplicon based studies. Amplicon data should, due to the short sequence fragments obtained by Illumina sequencing, just be used for a genus based assignment of the gut microbiota.

The comparison between the available 16S rRNA gene sequences of bacteria cultured by Callegari et al. (2020) and Tegtmeier et al. (2021), showed that for some genera different phylotypes were isolated in the different studies (Supplementary Fig. S4). According to the phylogenetic tree (Supplementary Fig. S4), more than one phylotype of *Providencia* was identified in our and other studies. The detection of different *Providencia* spp. was also shown for the gut of *Diptera* spp. (Kuzina et al. 2001; Toth et al. 2006). The reason for the diversity of gut associated *Providencia* species is not yet known.

The detected members of the *Enterobacteriaceae* clade represents a high number of possible species, assigned to the genera *Klebsiella* and *Enterobacter*. A distinction of members of this clade requires at least a multilocus sequence analysis (MLSA) or a core genome based phylogenetic analysis (Glaeser and Kämpfer 2015; Adeolu et al. 2016). Comparison of *Enterobacteriaceae* clade isolates of this study and those obtained by Callegari et al. (2020) and Tegtmeier et al. (2021) indicated a high diversity of different *Enterobacteriaceae* clade bacteria present in the guts of BSFL already by the comparison of the 16S rRNA gene sequences.

Species and strain specific variations were found in the efficacy to support BSFL growth and substrate utilization efficiency (Yu et al. 2011; Mazza et al. 2020). Our study showed that for several genera different phylotypes (potentially different species) and genetically different strains of the same phylotype (different genotypes) could be isolated from BSFL gut samples dependent on the applied cultivation strategies. Due to the limitation of the applied genomic fingerprinting PCR techniques to intra-lab comparison it is not possible to compare isolates from different studies to check if genetically identical strains were present in the guts of BSFL in different BSFL studies.

Several of the detected bacteria were closely related to bacterial pathogens. Cultured representatives are required for species assignment which is a requirement for risk group assessment. Further on isolates can be screened for pathogenicity factors or used for infection experiments to understand if BSFL associated strains indeed represent pathogens or just phylogenetically related to pathogenic strains.

As Gold et al. (2020) pointed out, little is known about specific functions and evolutionary adaptation of the BSFL gut microbes. In vitro studies are suggested to get more knowledge on the activities of those bacteria. Cultivation of gut and residue microbes is a pre-requisite for such studies. Large strain collections of gut microbes are required to study species and strain specific traits in the rearing process (Gold et al. 2020). Currently there are no genome sequence based studies of BSFL gut microbes available, which would explain either the diversity or the common shared genes of BSFL gut associated taxa, in general or of specific genera. It may be possible that gut associated strains share specific gene sets which can explain their adaptation to BSFL guts. That phenomenon was shown for *Aeromonas* strains, which occur as one of the two dominating gut symbionts in the gut of medicinal leeches (Marden et al. 2016; Ott et al. 2016).

## Conclusion

This study confirmed the findings of previous studies that few taxa are repetitively detected by the analysis of the gut microbiota of BSFL which seemed to be members of a core gut microbiota. The strain level based resolution performed in our study by genomic fingerprinting of isolated gut bacteria indicated, that the diversity of the gut microbiota within the abundant taxa is much higher than indicated by the culture independent 16S rRNA gene amplicon sequencing studies. Our data enlarge the knowledge of the intra-species diversity for BFSL gut bacteria, as it has been reported before for other insect gut microbiota (Ellegaard and Engel 2016).

The application of different media and different cultivation strategies has extended the genetic diversity for different taxa. Especially the dilution-to-extinction cultivation, originally developed for the cultivation of abundant slow growing marine oligotrophs (Button et al. 1993), extended the diversity of cultured bacteria for BSFL gut microbes. If there are specific strains within the abundant taxa that show different growth behaviours and were therefore just cultured by the dilution-to-extinction approach must be further studied.

Strain collections are the first step to move on to explore specific functions and the evolutionary adaptation of the cultured symbionts of the insect gut. Comparative genomics of cultured BSFL gut bacteria with representatives of the same taxa from other insect guts or free-living or even pathogenic strains must be the next step forward to determine genomics traits linked to functional traits as metabolic capacities and evolutionary adaptation to the specific ecological niches as the larval gut.

## Supplementary Information

Below is the link to the electronic supplementary material.

Supplementary file1 (DOCX 3256 kb)

## Data Availability

The Genbank/EBML/DDBJ of the 16S rRNA gene sequences generated from isolates are available under the accession numbers OK012097 to OK012261.
