# Supplementary material for: Isolation of Hermetia illucens larvae core gut microbiota by two different cultivation strategies
Source: Antonie Van Leeuwenhoek. 2022 Apr 22;115(6):821–37. doi: 10.1007/s10482-022-01735-7 (PMC9123031; doi:10.1007/s10482-022-01735-7)
Supplement: Supplementary file 1 — Supplementary file1 (DOCX 3256 kb) [file 10482_2022_1735_MOESM1_ESM.docx]

**Supplementary Figures**


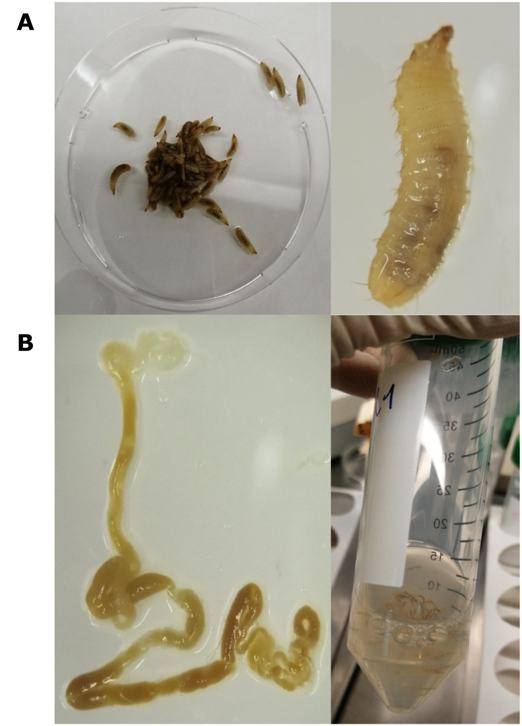


**Supplementary Fig S1.** **A.** BSFL before dissection. **B.** Dissected guts and resuspended guts in sterile 50 ml polypropylene tubes.
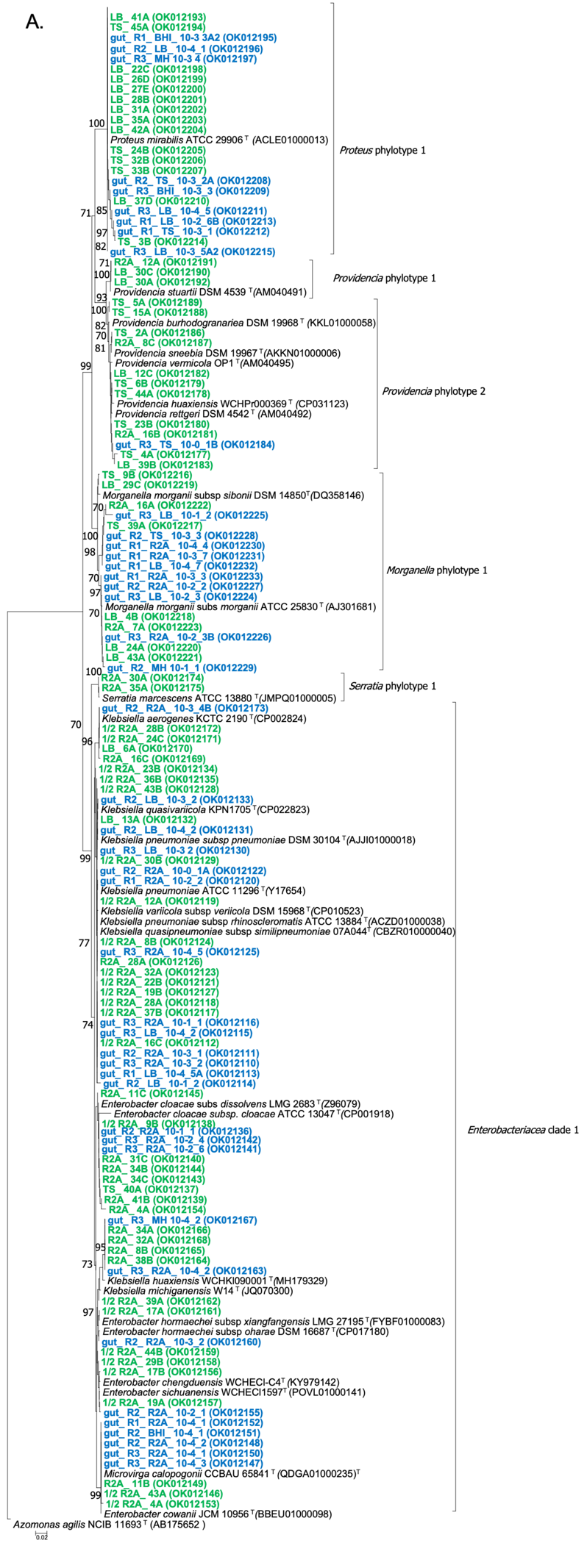

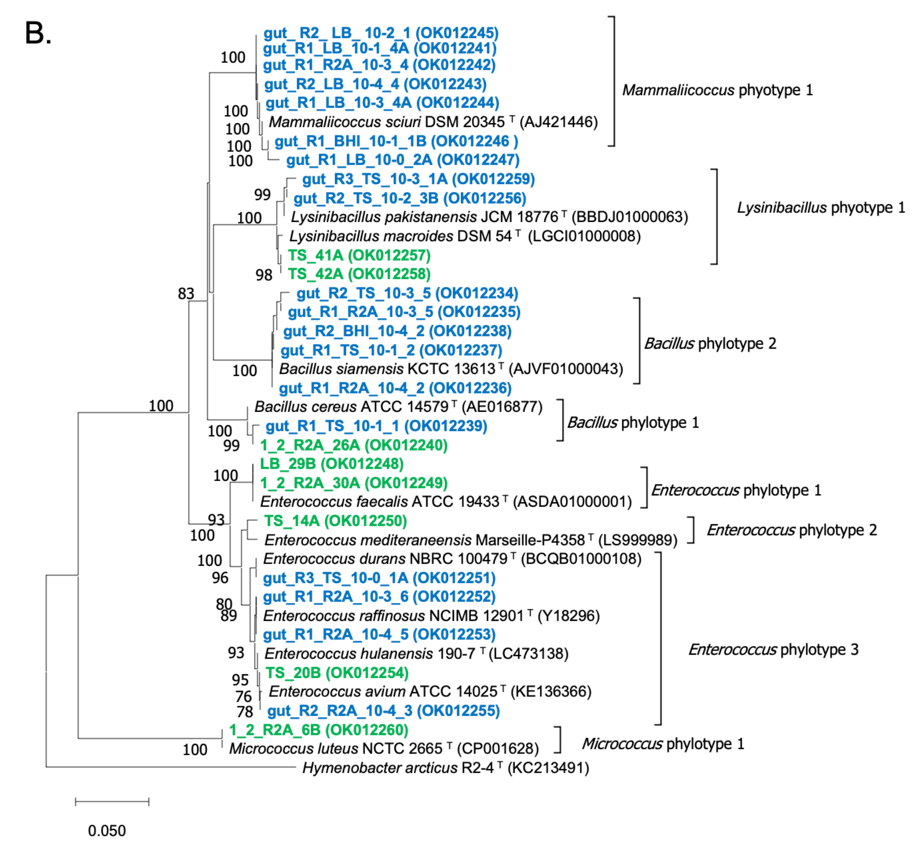


**
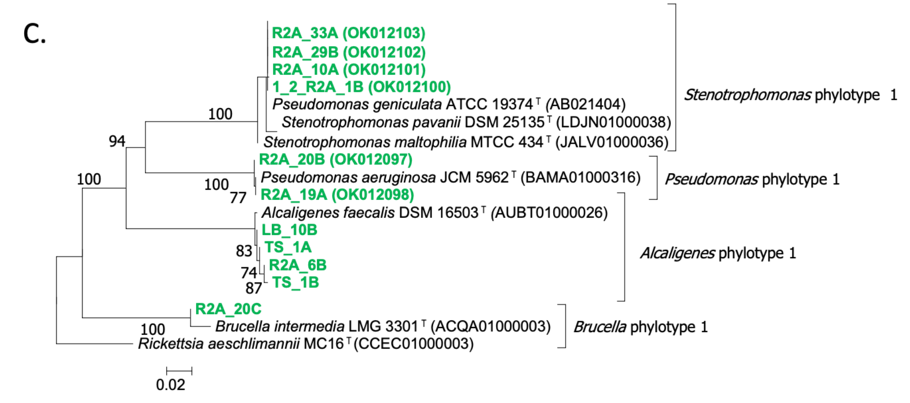
**

**Supplementary Fig S2.** Neighbour joining trees showing the phylotype assignment of the bacterial isolates cultured from BSFL gut samples based on partial 16S rRNA gene sequences. Trees were calculated for the *Enterobacteriaceae* clade (**A**), *Firmicutes* and *Actinobacteria* (**B**), and *Alpha*- and other *Gammaproteobacteria* (**C**). Analysis was performed in MEGA7 using the Jukes-Cantor distance correction as evolutionary model and 100 replications for bootstrap analysis. Bootstrap values (>70 %) are given at the branch nodes. All isolated culture by the direct plating are in blue bold and those from the dilution-to-extinction in green bold. Accession numbers of isolates and type strains are given in brackets.


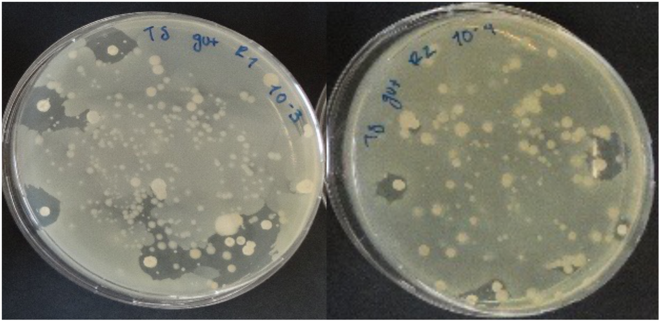


**Supplementary Fig S3.** Bacterial growth on TS agar after direct plating of serially diluted cell suspensions derived from the BSFL gut samples. An inhibition of swarming bacteria (identified as *Proteus* spp.) was obtained as clear inhibition zones around some colonies identifies as *Bacillus* spp.


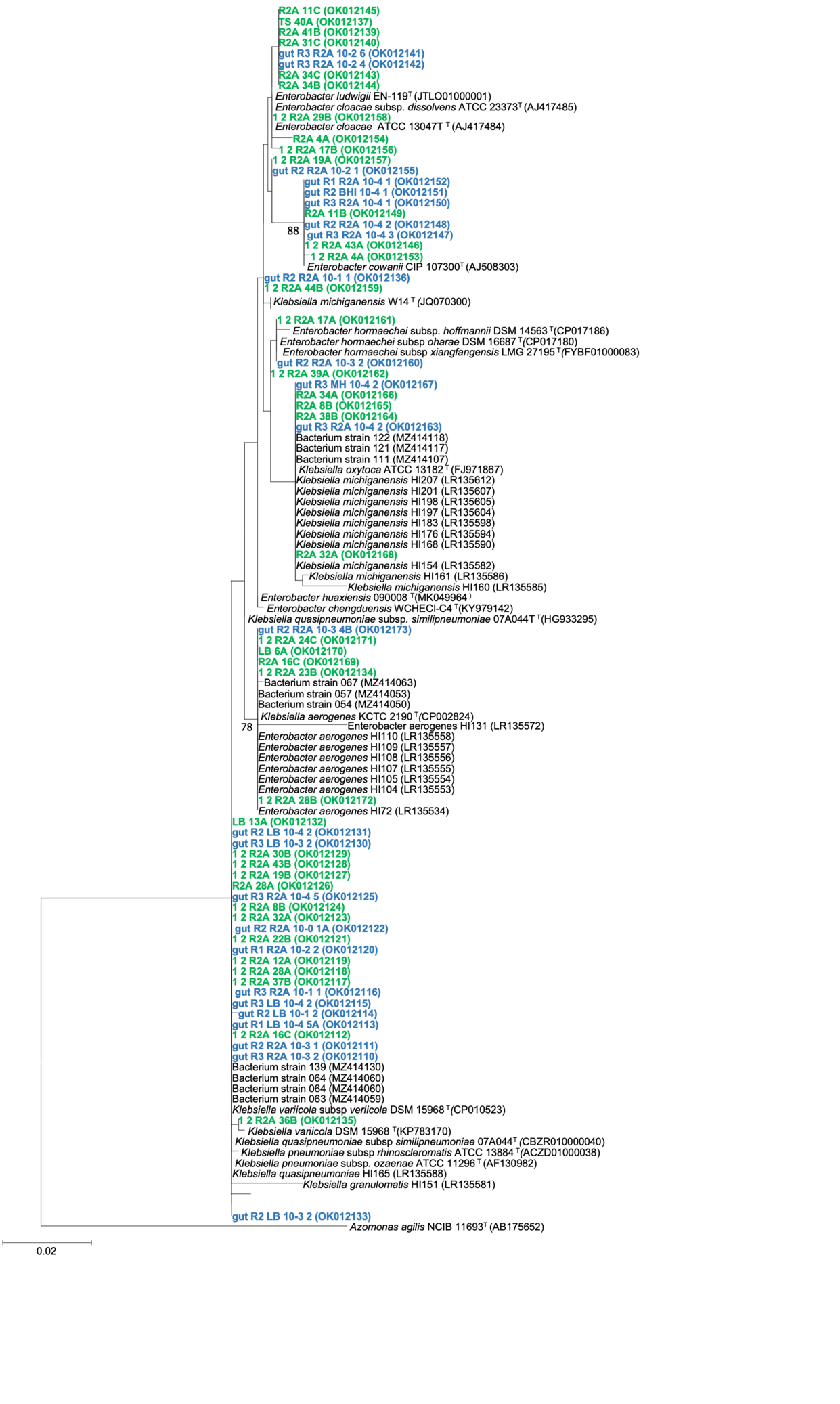


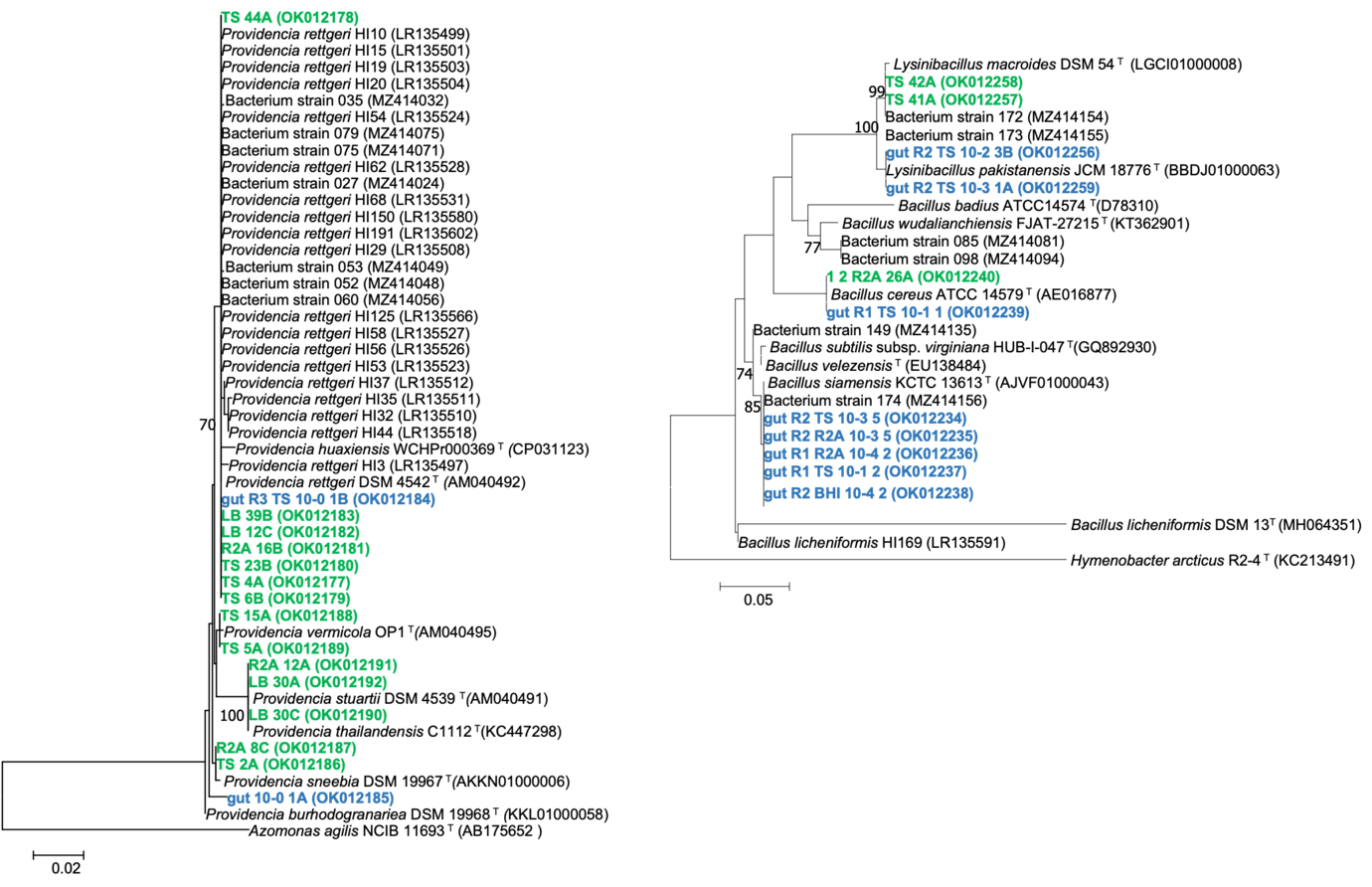


**Supplementary Fig S4.** Neighbour joining trees showing the phylogenetic placement of the bacterial isolates from Callegari et al. (2020), Tegtmeier et al. (2021), and this study from BSFL gut microbiota. Trees were calculated based on partial 16S rRNA gene sequences in MEGA7 using the Jukes-Cantor distance correction as evolutionary model and 100 replications for bootstrap analysis. Bootstrap values (> 70 %) are given at the branch nodes. All isolates from this study are in blue bold for direct plating and green bold for dilution-to-extinction. Accession numbers are given in brackets.
